# Induction treatment of acute myeloid leukemia in an elderly patient with intramarrow injection/administration of cytarabine. Second case report

**DOI:** 10.1002/ccr3.1081

**Published:** 2017-07-28

**Authors:** Anwarul Islam

**Affiliations:** ^1^ Division of Hematology/Oncology Department of Medicine Buffalo General Hospital Buffalo New York USA

**Keywords:** Acute myelogenous leukemia (AML), cytarabine (Ara‐C), elderly patients, intramarrow injection/administration

## Abstract

We show for the second time that intramarrow injection/administration of chemotherapeutic agents such as cytarabine (Ara‐C) can be used safely and effectively and is associated with no toxicity, promising antileukemic activity and possible improved survival.

## Introduction

In recent years, there has been some considerable progress in the treatment of acute and chronic leukemia, lymphoma, and multiple myeloma in adults. The majority of the patients now achieve complete remission (CR) following chemotherapy. However, with the exception of some patients salvaged by bone marrow transplantation, the vast majority of these patients eventually relapse and succumb to their disease. Patients who relapse soon become resistant or refractory to repeat intravenous chemotherapy. Thus, treatment ultimately fails in a majority of patients and new approaches are necessary to improve the prognosis, quality of life and long‐term survival.

Current therapy of acute myelogenous leukemia (AML) has evolved over many years of research, experience and by trial and error. However, there has been very modest change in the strategies of treatment of this disorder except for the introduction of occasional new drugs, different dosage, and alternative dose schedules. Although the overall response rate to the so‐called standard (7 + 3) induction therapy for AML 1 coupled with refinements in supportive care has improved the CR rate 2, most patients do relapse and usually fail to undergo further remission. Furthermore, elderly patients with AML offer a unique therapeutic challenge as they cannot tolerate intensive chemotherapy 3, 4, 5 and they also display poor prognostic characteristics such as frail constitution, adverse cytogenetics, and a myriad of comorbidities 6. Innovative new approaches are thus necessary to treat newly diagnosed elderly patients with AML 7, 8, 9 with a view to control the disease, improve the quality of life, and afford progression‐free survival. With this view in mind, a newly designed approach of “intramarrow injection” therapy has been developed using low dose Ara‐C to treat newly diagnosed elderly patients with AML which is seen to offer some encouraging results 10. Here, we report another elderly patient with AML who has been successfully treated with intramarrow injection of Ara‐C.

## Case report

The patient, an 85‐year‐old white male with past medical history significant for colon cancer, status post polyp removal and subsequent partial colectomy, no chemo or radiation therapy, history of deep venous thrombosis (DVT) and pulmonary embolism (PE) following his colectomy, IVC filter placement, and history of prostate cancer untreated, history of chronic kidney disease presented to the emergency room of hospital A with 2 days history of chest pain, generalized weakness and mild shortness of breath. Lower extremity Doppler study revealed nonocclusive DVT of the right mid‐ and distal superficial vein and right popliteal vein. The patient was put on heparin and was admitted to intensive care unit. A 2D echo showed a small pericardial effusion and a normal ejection fraction; however, a CT of the chest without contrast demonstrated moderate to large pericardial effusion and bilateral pleural effusion. Troponin was negative, but prostate specific antigen (PSA) was elevated at 17.

On physical examination the patient was noted to be anemic, but he was not in acute distress. There were no jaundice, cyanosis, or edema. His abdomen was soft and nontender. Bowel sounds were heard. Liver, spleen, and kidneys were not palpable. There was no palpable lymphadenopathy. Heart sounds S1 and S2 were identifiable along with a soft ejection systolic murmur. Chest examination revealed a few scattered rhonchi and diminished breath sounds at the bases. His vital signs were stable, and the patient was afebrile.

**Figure 1:**
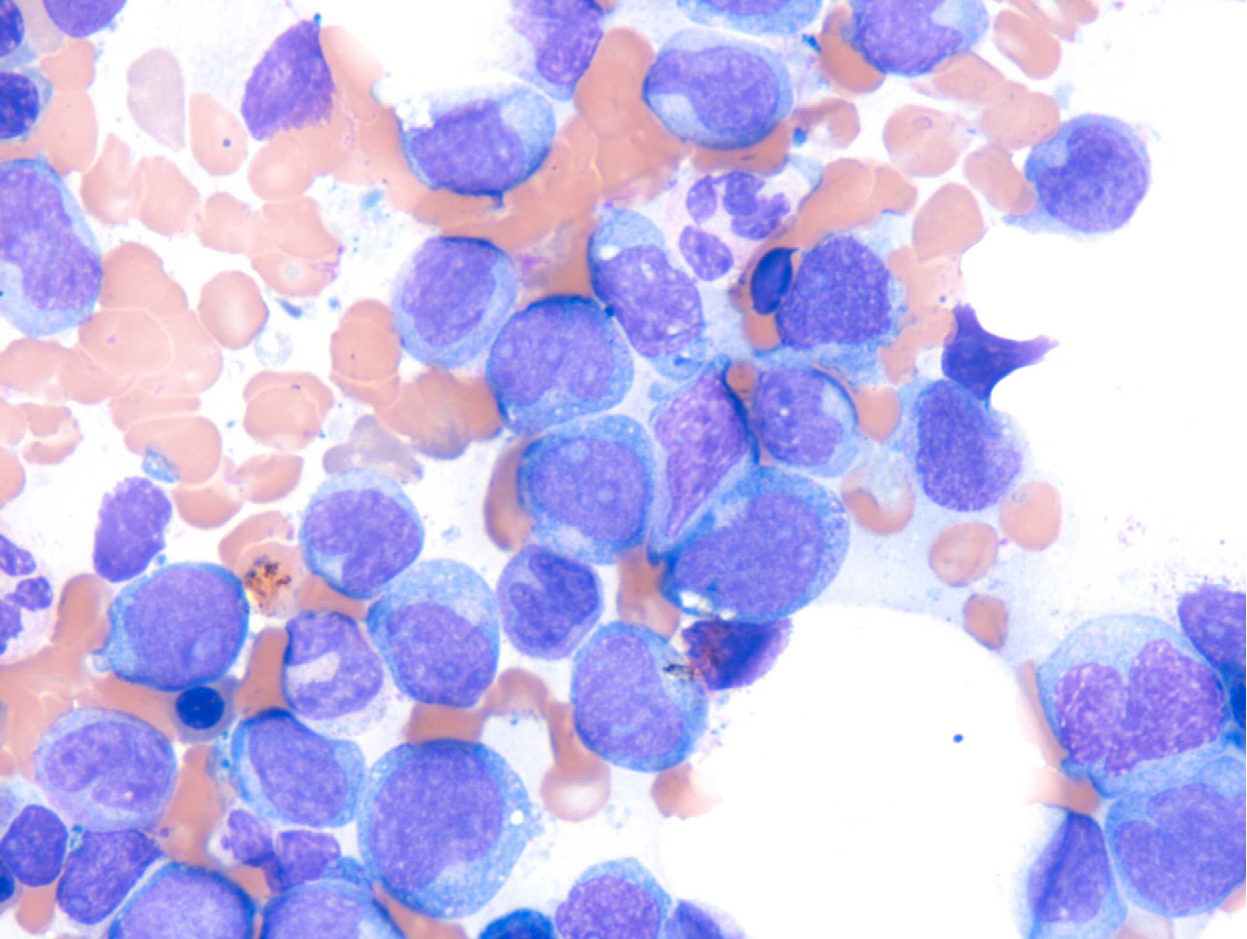
Bone marrow aspirate smear showing immature myeloid cells (myeloblasts and monoblasts).

Laboratory investigations revealed WBC 15.6 × 10^9^/L, hemoglobin 6.7 g/dL with normal MCV, and MCH and a platelet count of 82 × 10^9^/L. A manual differential of his peripheral blood smear revealed 10% myeloblasts, 17% monoblasts, and 15% promonocytes. A bone marrow aspirate and biopsy revealed a hypercellular marrow (80%) with frankly leukemic picture with more than 30% blast cells. Morphologically, the blast cells appeared to be a combination of myeloblasts (14%) and monoblasts (23%) (Figure 1). Monocytes and promonocytes constituted about 24% of the hemopoietic cell population and plasma cells were prominent (5%).

**Figure 2:**
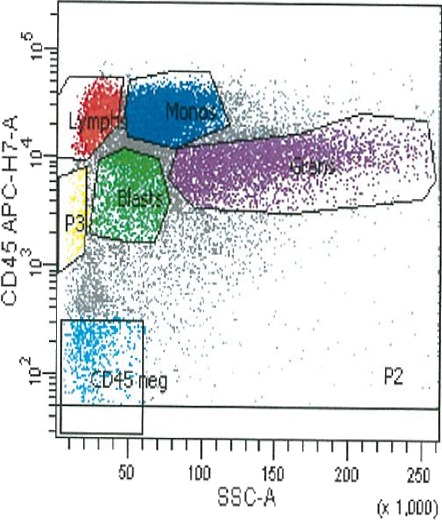
Cluster analysis of blasts using CD45/side scatter characteristics. Green, blasts; blue, monocytes; red, lymphocytes; purple, granulocytes.

Flow cytometry studies of the bone marrow aspirate sample revealed an abnormal blast cell population (Figure 2). The blast and monocyte gate contained 50% of the total events which consisted of two populations. One population comprised of 2% of total events positive for CD34/CD117/CD13/CD33/HLA‐DR consistent with myeloblasts. The other population was positive for HLA‐DR/CD13/CD33/CD64/CD14/CD15 and dim CD56 while negative for CD34/CD117 consistent with a monocytic population with aberrant CD56‐population. Based on the CD45 vs SSC characteristics, the mature myeloid form showed decreased side scatter expression, consistent with hypogranularity. The plasma cell gate contained 0.3% of total events which consisted of a population of CD19/CD38/CD138‐positive polytypic plasma cells with kappa:lambda ratio of 1.4:1. From the above investigations, a diagnosis of acute myeloid leukemia with monocytic differentiation and slight plasmacytosis was made.

The patient also underwent thoracentesis, and approximately 400 mL of cloudy yellow colored fluid was removed. One cytospin slide and one direct smear showed numerous monocytes with immature forms in a background of blood and few reactive mesothelial cells. These cytologic features were considered to be consistent with involvement by the patient's recently diagnosed acute myeloid leukemia (AML) with monocytic differentiation.

Cytogenetic studies: a total of 20 metaphase spreads were analyzed by G‐banding, which revealed a normal male karyotype of 46, XY. No apparent clonal chromosomal aberrations were identified. FISH analyses with the AML panel probes (t[8;21], 11q23, t[15;17], 16q22, 17q21) were performed and showed no aberrations. In addition, FISH analyses with the probe specific for ABL1/BCR fusion showed no PH^1^ rearrangement.

After providing informed consent, the patient was treated with intramarrow injection of Ara‐C. The patient was premedicated with 100 mg of hydrocortisone and 50 mg of Benadryl intravenously half an hour before the intramarrow injection of Ara‐C. The first injection of Ara‐C (50 mg/m^2^) was given into the right posterior ilium. The subsequent intramarrow injections of Ara‐C (30 mg/m^2^) were given into the sternum (each time a slightly different area of the sternum was chosen) on 3 days a week for 2 weeks. The patient tolerated the treatment procedure well and without any untoward affects, particularly nausea and vomiting which commonly occurs during Ara‐C infusion.

Five days following the start of the treatment, his WBC count fell to 6.3 × 10^9^/L and the leukemic cell counts fell to 40% (myeloblasts (8%) and monoblasts (15%), promonocytes 10%, and monocytes 7%). Plasma cells remain prominent (5%). His platelet count remained stable (80 × 10^9^/L), and at this stage the peripheral blood smear also presented a few mature granulocytes.

The patient thus showed a dramatic response to intramarrow injection of Ara‐C particularly with respect to a rapid elimination of blast cells from the peripheral blood and perhaps also from the bone marrow. In addition following 2 weeks of intramarrow injection of Ara‐C, the patient's pleural effusion started to regress. The patient remained effusion‐related symptom‐free for 6 months. Furthermore, there was also a complete regression of his pericardial effusion as evidenced by CT scanning.

**Graph 1:**
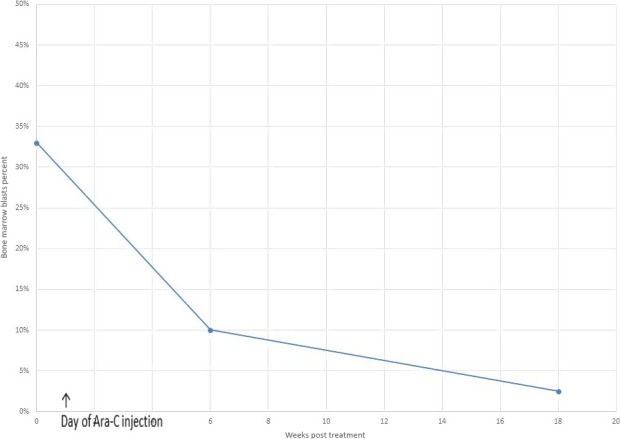
Effect on bone marrow blast cells following intramarrow injection of Ara‐C.

A BM aspiration and biopsy conducted about 6 weeks following intramarrow injection chemotherapy showed a slightly hypercellular marrow with markedly reduced blast cell population (10%) (Graph 1) and promonocytes and monocytes constituted 27% of total nucleated cells. Polytypic plasma cells remained increased (7–10% of marrow cells). Fluorescence in situ hybridization (FISH) analysis performed on the bone marrow specimen using DNA probes for MDS and AML did not reveal any specific abnormalities.

**Graph 2:**
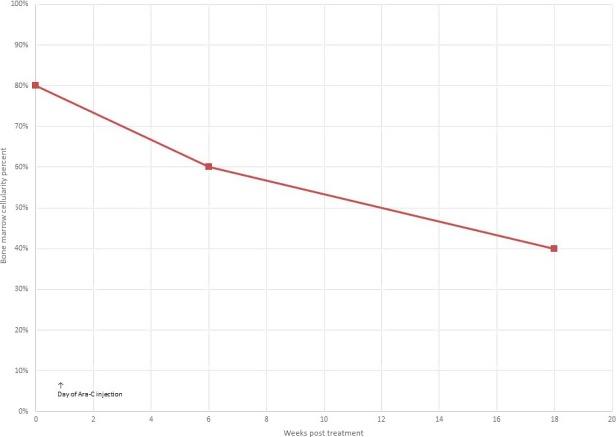
Effect on bone marrow cellularity following intramarrow injection of Ara‐C.

A BM aspiration and biopsy performed 18 weeks following intramarrow injection chemotherapy showed a normocellular marrow (Graph 2) with mild dysmegakaryopoiesis and plasmacytosis. Kappa and lambda immunostains did not show any obvious light chain restriction. Blast cells were 2.5% and promonocytes 1%.

The patient thus showed a dramatic response to intramarrow injection of Ara‐C particularly with respect to elimination of blast cells from the bone marrow and peripheral blood. The patient continued to show clinical improvement except for the fact he remained mildly leucopenic and thrombocytopenic even though his marrow had become normocellular and showed trilineage hematopoiesis with some megakaryopoietic dysplasia. Unfortunately, he became transfusion dependent. Due to that, his advanced age and as he became unable to perform the activities of daily living, his family decided against any continued supportive therapy (in the form of blood transfusion) and opted for hospice care. The patient expired a few days after he entered into the hospice environment.

## Discussion

During conventional chemotherapy, chemotherapeutic agents are typically injected intravenously. Intravenous injection, however, as a method of chemotherapy, has a number of potential disadvantages. These include delivery of potentially toxic agents to nontarget regions of the body and a requirement for high doses to overcome the effects of dilution, metabolism, and degradation of the drug(s) throughout the body. Therefore, the injection of a drug directly into a target region (in this case bone marrow) affected by the disease is desirable.

A number of diseases afflict the bone marrow specifically. These include leukemia, lymphoma, and multiple myeloma. These cancers are typically treated by intravenous chemotherapy. The intravenous mode of delivery suffers from the limitations cited above. Vascular injection results in only a small percentage of the agent reaching the target organ as in the case of the marrow in AML. Thus, there is a need for improved devices and methods for effectively injecting therapeutic agents directly into the marrow to maximize the therapeutic effect and minimize the toxicity with relatively smaller dose being used which might be potentially very useful for elderly patients with AML.

Research conducted by the author using the plastic embedded (processing method) bone marrow sections 11 resulted in the observation that the interface between the solid bone (bony trabeculae) and bone marrow was not separated but remained intact. The endosteal cells which line the bony surfaces were more readily identifiable and did not become deformed or displaced as they would have been if conventional paraffin embedding had been utilized. Analysis of these technically superior bone marrow sections which provided an improved details of cell morphology in the bone marrow similar to what is observed in vivo showed for the first time an implication of the endosteal region in the origin and the spread of leukemia 12, 13.

Based in part on observations of the endosteal cells in plastic embedded bone marrow biopsy (BMB) sections from normal adults and patients with various hematological disorders such as leukemia, lymphoma, multiple myeloma, aplastic anemia, and myelodysplastic syndrome 11, 12, 13, 14, 15, it was postulated that the endosteal cells are reminiscent of embryonal stage mesenchymal cells and, depending on the needs of the body, may differentiate into myeloid, lymphoid, stromal or fat cells. In leukemias, particularly in AML, it was observed that the leukemic blast cells were originating from the endosteal region. In some instances, the endosteal cells appeared to be giving rise to the leukemic blast cell population 12, 13. These observations prompted investigations into the potential treatment of leukemia through injection of chemotherapeutic agents directly into the marrow cavity 10 whereby chemotherapeutic agents not only affect the leukemic cell population in the intertrabecular marrow space where leukemia proliferates but also affect the endosteum where leukemia, according to the hypothesis, originates 13.

When conventional intravenous administration of chemotherapeutic agent is employed, the drugs reaches the leukemic population in the marrow at a diminished concentration as it is immediately diluted in 5 L of blood and its effectiveness is also mitigated by other ambient factors discussed above. Importantly, the drug/chemotherapeutic agent may possibly never reach the endosteum where leukemia is proposed to originate. Thus, direct intramarrow injection of chemotherapeutic agents could potentially improve the delivery of the drugs to the essential (target) cells, thereby improving the response and outcome of therapy for such patients as observed in the case presented.

Although an injection of chemotherapeutic agents into the hip bones or sternum may not directly affect other bone marrow regions, the agents released here are absorbed by venous sinusoids of this highly vascularized organ and ultimately reach distal bone marrow and other regions almost as effectively as by the conventional (intravenous) route of administration. Although the plasma levels of Ara‐C were not measured after its intramarrow administration, the gradual reduction in the pleural effusion and regression of pericardial effusion demonstrate that significant amounts of the drug were also absorbed and reached the circulation in an active form. Thus, the therapeutic effect of Ara‐C in this case may not only be due to destruction and killing of the leukemic cells in the ilium and sternal marrow but most likely to have affected the leukemic cell population in other bone marrow regions as well as affecting the leukemic cells in the pleural and pericardial cavity. A similar effect has also been reported in a case with non‐Hodgkin's lymphoma following intrapleural instillation of rituximab 16.

An additional therapeutic benefit of intramarrow injection may result from the induction of necrosis within the marrow caused by the relatively high concentration of a chemotherapeutic agent delivered directly in the marrow cavity. The delivery of the drug at a high concentration at the target site would result in destruction of malignant cells, induce necrosis, and thereby summon macrophages to the site of destruction of the leukemic cells. This localized process may also affect the microenvironmental cells that control hematopoiesis and immuno‐regulatory cells. The overall combined effects may initiate a beneficial outcome as observed in a chronic lymphocytic leukemia (CLL) patient following spontaneous bone marrow necrosis 17. Research in the last few years has revealed a sophisticated interaction between the bone marrow stromal cells (microenvironmental cells) and myeloid malignancies 18.

As the title of this report indicates this is the second case in which we have used intramarrow administration of low dose Ara‐C in the treatment of an elderly patient with AML. There are no other clinical and morphologic studies assessing intramarrow injection of chemotherapeutic agents nor are there any specific comparative studies between a standard Ara‐C dose and the reported schedule.

This report considers the second patient where direct intramarrow injection therapy with Ara‐C was used and this strategy seems to have worked as it did in the previous case 10. In this particular patient, the beneficial results are evidenced by the fact that the leukemic blast cells were significantly reduced in number in the peripheral blood within days of starting the therapy and the appearance of mature neutrophils and reduced number of monocytes in the circulation. Clinically, the patient also felt better and nausea and vomiting were absent. About 3 months following his intramarrow injection therapy with Ara‐C, the patient became increasingly transfusion dependent requiring 1–2 units of blood transfusion almost every 2 weeks. The reason for this was not entirely clear. It is generally believed that the patients who become transfusion dependent may have more aggressive disease biology 19, but this was not borne by the fact that his cytogenetics, as well as molecular studies, was all normal.

It is understood that a standardization of response criteria and treatment outcomes is required for the proper evaluation of treatment protocols 20. The brief and limited study of our patient prevented this type of assessment. Prospective randomized clinical trials are warranted to compare other conventional therapies such as low dose Ara‐C alone versus LDAC in combination with all‐trans retinoic acid (ATRA) and/or arsenic trioxide (ATO) in order to clarify the role of such treatments in elderly patients with AML 21.

In our experience, the management of older patients with AML has been difficult and challenging as older patients have multiple comorbidities. They are either unfit, vulnerable, or too frail to withstand intensive chemotherapy 22. In elderly patients, the disease also tends to be more resistant to chemotherapy 23 and frequently present with unfavorable AML subtypes 24. Other factors such as molecular or cytogenetic abnormalities associated with poor clinical outcome are more often detected in elderly patients 2, 25. Although treatment of elderly patients with AML has increased over time, in a large observational study of Medicare beneficiaries have shown that currently about 50% of the elderly AML patients remain untreated following diagnosis 26 because of the fear of treatment‐ related mortality and resistance to therapy 27. The best treatment protocol for older patients with AML remains to be identified, and in that respect, we believe that this new treatment protocol with intramarrow injection of chemotherapeutic agents like Ara‐C may fulfill an unmet need.

## Conflict of interest

The authors have no conflict of interest.

## Authorship

AI: Treated the patient and wrote the manuscript.
